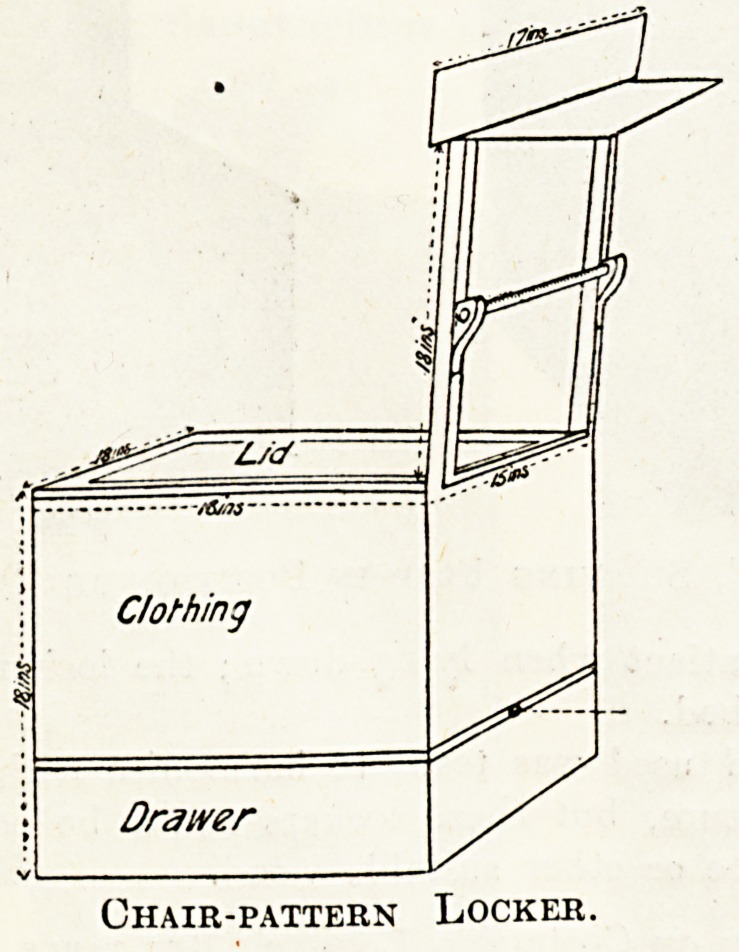# Suggestions for an Ideal Locker: The Six Selected Designs

**Published:** 1914-05-09

**Authors:** 


### The Six Selected Designs.

The following are the six designs which have been selected as being the best of the numerous descriptions which have reached us for this competition. The order in which they appear is no indication as to the relative merit of each, and all the descriptions and illustrations should be considered carefully and judged independently by our readers, who should then record their votes on the form given on p. 164.

### Locker A.

**Small and Cheap—Thirty Shillings.**

The three photographs represent a locker made for the Grimsby and District Hospital by a local firm. The wood is hard oak, well seasoned, stained and polished to match ward floors and furniture. The height is 28½ in. It has a drawer 6½ in. deep, 1 in. in thickness, and a plain oak knob. Below is a cupboard divided into two parts by

Front Views of Locker A.

a loose shelf, ½ in. thick, which rests on two ledges, one each side of locker. The door is fastened by a ball catch and opens by means of a small knob. The locker runs on four brass castors.

It has a towel rail at the back, standing out 2½ in., made of oak, fitting into lacquered supports.

The top of locker is of polished oak with moulding round, into which fits a piece of plate glass, 15 in. by 17 in. This can be removed for cleaning underneath by turning locker on to its side.

The chief merits are: (1) cheapness (30s. contract); (2) durability; (3) good workmanship; (4) towel rail, wide enough to hold thick towel; (5) loose shelf, which can easily be removed and thus permits of thorough cleansing

Side View of Locker A.

of cupboard; (6) moulding round top of locker to protect edges and corners of glass.

The demerit is the glass top. Hot fluids might crack it and get underneath. Dust may collect, but can be overcome by taking out glass. The glass top can be renewed at a cost of 2s. 3d.

Tops made of marble, tiles, or enamelled iron, etc., could be used instead of glass, but would cost more.

### Locker B.

**A Metal Locker.**

When choosing a locker it is better to know what to avoid rather than what to adopt; the accompanying photographs will serve as an illustration.

This locker (Norfolk and Norwich Hospital) is formed of tubular-iron standards, with sheet-iron bottom and sides, and is treated throughout with metallic paint.

There is no advantage in the rubber-tyred castors; the locker is removed such short distances usually that there is no real necessity for it to be noiseless, so that an all-iron castor would possess the advantages of being cheaper, permanent, and admit of being cleaned more easily.

The front, by opening in the manner shown, and supported by chains, is a convenience to the patient when obtaining anything from the locker. The flanged drawer is carried on angle iron. The top is of plate-glass, obscured on the under side, and resting on four rubber stops; this has certain disadvantages: it adds considerably to the initial cost and to the upkeep by making the locker top-heavy, so that in the event of any sudden tilt

Locker B, Front and Side Views, Showing Let-down Flap.

while being moved there is nothing to prevent the glass from falling off, and in addition there is the constant risk of some hot utensil being placed on it, when it breaks instantly.

The plated caps to the standards require daily polishing, and are a disadvantage. There would be no need for rubber stops, plate-glass, or plated caps if the top were formed in one with the sides and back in sheet iron and so consolidate the whole. The measurements are: Height, 2 ft. 9 in.; width, 1 ft. 4 in.; depth, 1 ft. 4 in.; locker, 9 in. high; drawer, 3½ in. deep; castors, 2 in. deep.

### Locker C.

### A Sanatorium Locker.

Locker C, Open and Closed.

We are supplying in the Daneswood Sanatorium a set of lockers designed partly on the pattern called the "Cromer" shown in The Hospital. Patients who are cabinet-makers by trade are making them, so that the cost in labour is nothing, and they are in every way attractive and strong. They are made of American white wood, polished to resemble walnut, have white tiled tops, and are fitted with wooden towel rail at the back and stand upon block feet with castors. Their height is 3 ft. 6 in.; their breadth and depth 18 in. They contain two shelves and their cost is 19s. each, made out as follows: Best white wood, 14s. 6d.; castors, 1s. 6d.; hinges, screws, and fastener, 1s.; nine tiles, 1s. 6d.; polish, glue, and sandpaper, 6d. Our previous lockers were enamelled iron, but these, by their great tendency to rust, are very unsuited to open-air institutions.

### Locker D.

### In Use at a Consumption Hospital.

This locker is in use at the new Great Barr Park Hospital for Consumption. It was specially designed for us according to my ideas of a useful and

Locker D, Showing Flap Extended and Drawer Open.

aseptic piece of furniture. It is iron, but, unlike the usual locker (white enamelled), this one is painted with aluminium stoved to render the sur-

Locker D, Back View.

face hard and then burnished. There is a divided drawer and cupboard, towel rail at back, two plate-glass shelves, and large rubber-tyred castors. Size of locker, 16 by 16 by 32. At the side is an adjustable bed-table, also in iron, aluminium painted, which can be raised to suit any height of bed. When not in use this table folds away. The legs on the side opposite the bed-table are weighted with lead. In this way, when dishes are resting on the table, the locker is evenly balanced. A locker of this description is sufficient for the needs of the patient. Our beds are always wheeled out on the verandah during suitable weather, and having large castors the lockers can easily be wheeled out too. Aluminium paint gives light and brightness to the wards; it withstands the open air well. Unlike white enamel, it does not chip, and should it get scratched a mere novice can touch it up with aluminium paint to look as good as new.

Our lockers were supplied by the Surgical Manufacturing Company, 85 Mortimer Street, London, and cost £2 9s. each.

### Locker E.

### A Small Infirmary Locker.

I enclose sketch to scale and a photograph of the locker in use at the North Devon Infirmary in the adult wards.

Locker E, Showing Front and the Ventilated Back.

It has a white marble top, a drawer with brass handle, a cupboard (ventilated behind) with one shelf; there is a

Locker E, Showing the Bed-table in Use.

brass towel rail and a wooden hollowed tray for soap, also two brass hooks, one for small enamelled mug to use when brushing teeth and the other for washing flannel.

Sectional Sketches of Locker E (Scale One Inch to the Foot.)

There is a folding flap table, which is used at meals, for reading, and writing. The locker was designed by the matron and supplied by the Ladies' Association. This locker has been in use for about seven years.

### Locker F.

### A Combined Locker, Bed-Table, and Seat.

Fifty-two of these lockers have recently been made for the Coventry and Warwickshire Hospital. Before adopting the final design we had a rough sample made in deal, which was modified and altered considerably to suit our requirements, and so admirably does the locker fulfil its treble purpose that I am unable to suggest an alteration which would improve the design. Its height is, of course, governed by the height of the bed, as the extending flap F should just

Locker F, Showing Flap in Position as Bed-table.

clear the patient when lying down; the locker part goes under the bed.

The wood used was teak, to harmonise with the other ward furniture, but these lockers could be equally well made in pine or other suitable wood.

Description of Combined Locker, Bed-table, and Seat.

A. Compartment for patient's linen, etc. When the locker is in use as a bed-table this part goes under the bed.

B. Hinged top to ditto, forming seat.

C. Flap, hinged to open outwards to allow of easy cleaning when shut, held in position by hooks LL.

D. Cupboard for soap, hair-brushes, etc. Door fixed by bullet catch.

E. Shelf in ditto.

F. Flap forming bed-table. When in position this is supported by hinges GG, and is of sufficient height just to clear the patient when lying down or sitting up.

SKETCH OF LOCKER F, SHOWING DIFFERENT PARTS.

GG. Brackets on hinges for ditto. These shut into a recess when flap F is down.

H. Back-board for resting chart frames, etc.

I. Small piece of beading to prevent slipping of charts, etc.

J. Towel rail at back.

K. Flap (F) in position as bed-table.

LL. Hooks inside locker to hold hinged flap C.

### A Chair-Pattern Locker Suggested.

A SEVENTH AND EXTRA DESIGN.

The compartment for clothing, with space for drawer included, is 18 in. in height, diameter, and width across the front, but behind is narrowed to 15 in. in width.

CHAIR-PATTERN LOCKER.

The top forms a lid. To allow lid, when open, to rest on back of chair, place hinges forward 1 in. from join of compartment with back of chair. The floor of compartment for clothing is placed 6 in. from the bottom, is made to slip into a groove which runs round inside on three sides, and is slipped in from behind, there being a knob which when pulled the floor is removed for cleaning. Underneath is a drawer 4 in. deep, opening in front, useful for things not advisable to be mixed up with clothing.

The height of the back of chair from level of lid of compartment to top is 18 in., and consists of two bars $1\frac{1}{2}$ in. broad, connected at top by a 3-in. board 17 in. long. This horizontal bar juts out 1 in. on either side beyond the width of back of chair. Fixed to it behind is a 7-in. shelf by 15 in., convenient on which to place cups and glasses. Twelve inches from top, and attached behind to the two perpendicular bars, is a towel roller; its two wooden rests jut out behind. The roller fits into a hole in the one rest and slips down into a semicircular groove in the other.

## Figures and Tables

**Figure f1:**
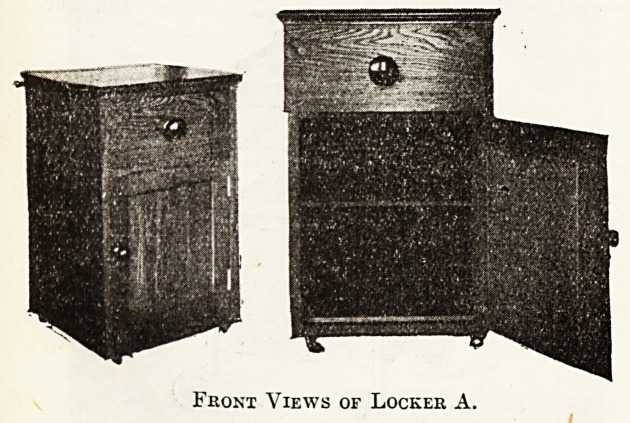


**Figure f2:**
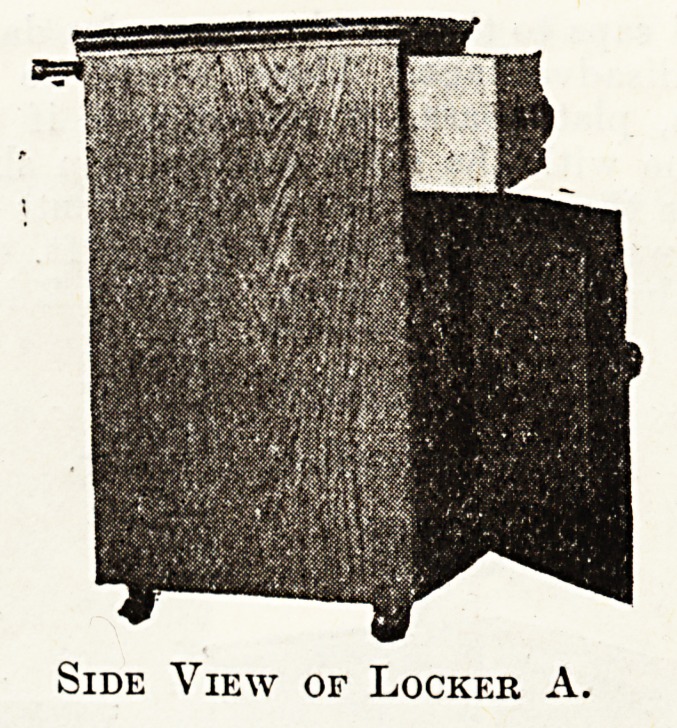


**Figure f3:**
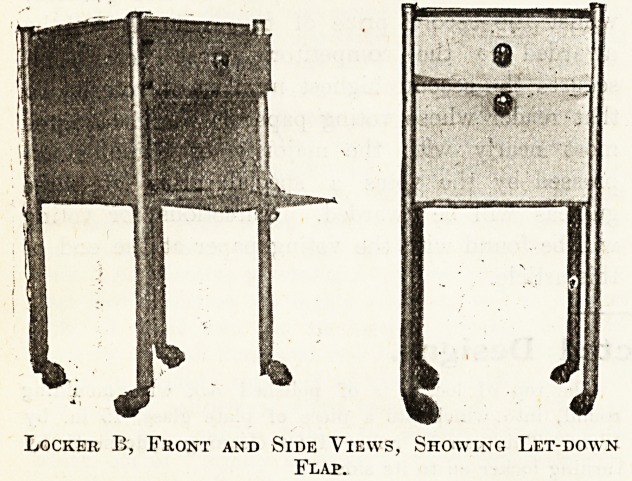


**Figure f4:**
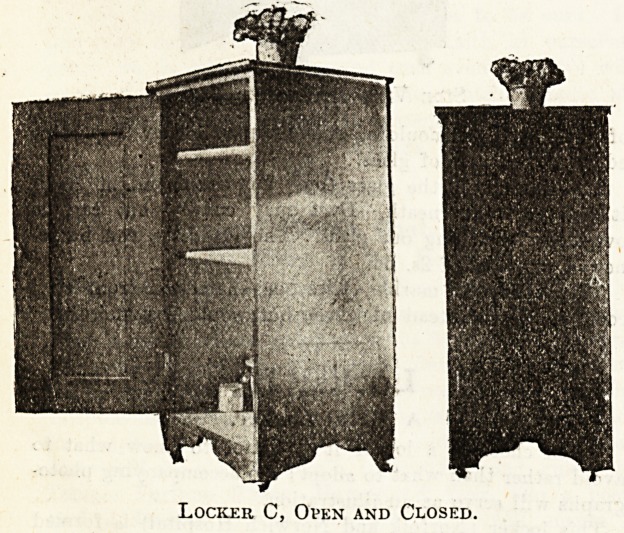


**Figure f5:**
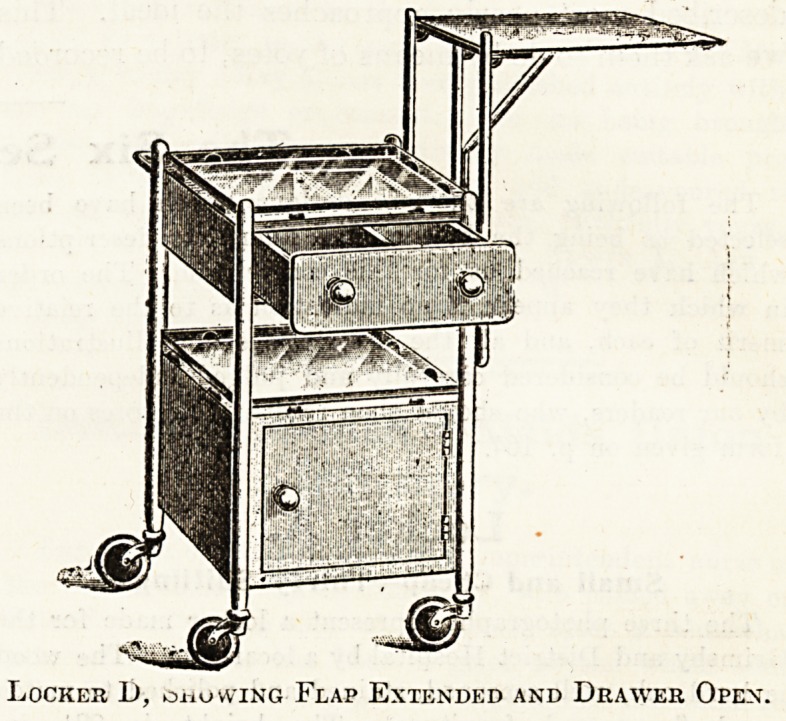


**Figure f6:**
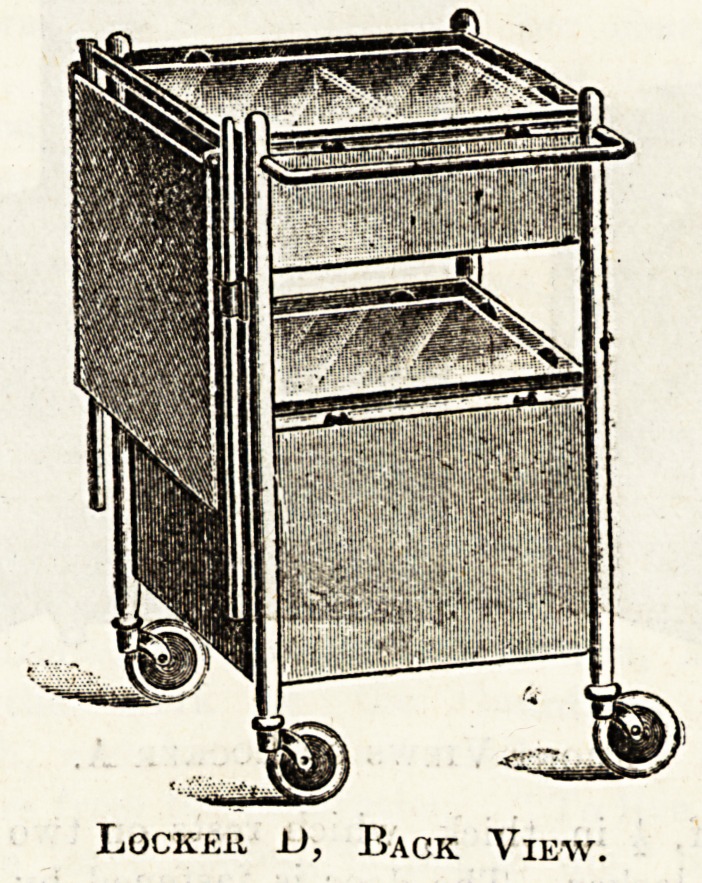


**Figure f7:**
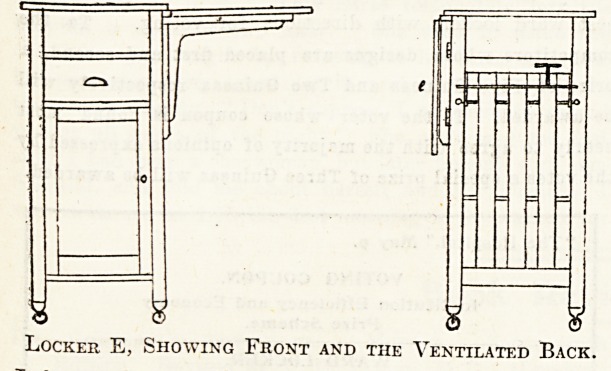


**Figure f8:**
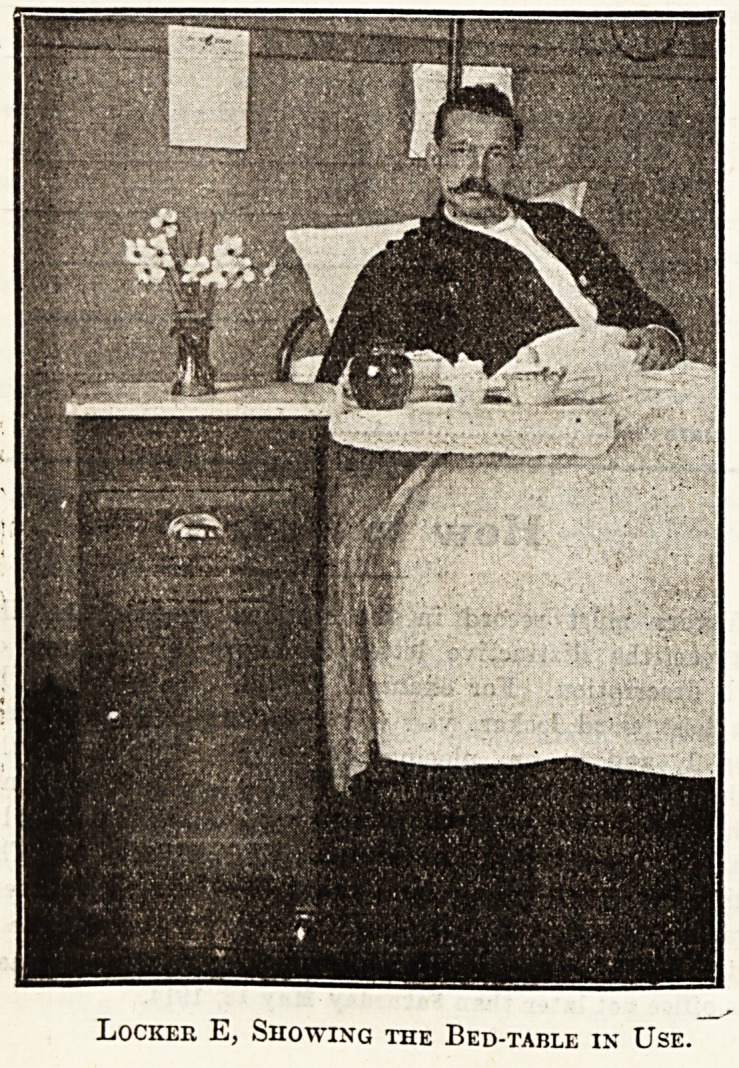


**Figure f9:**
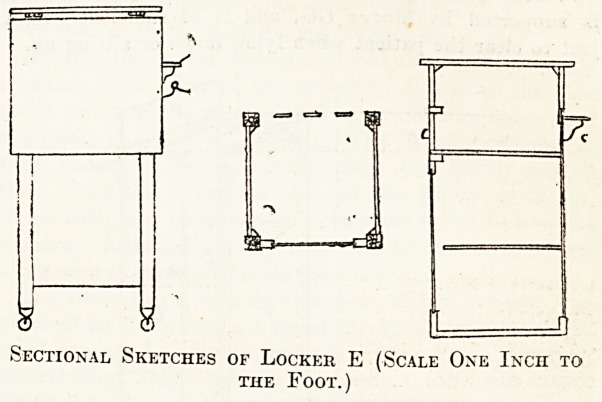


**Figure f10:**
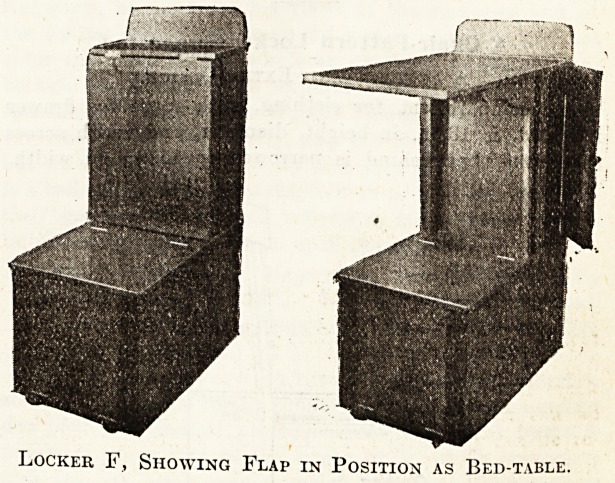


**Figure f11:**
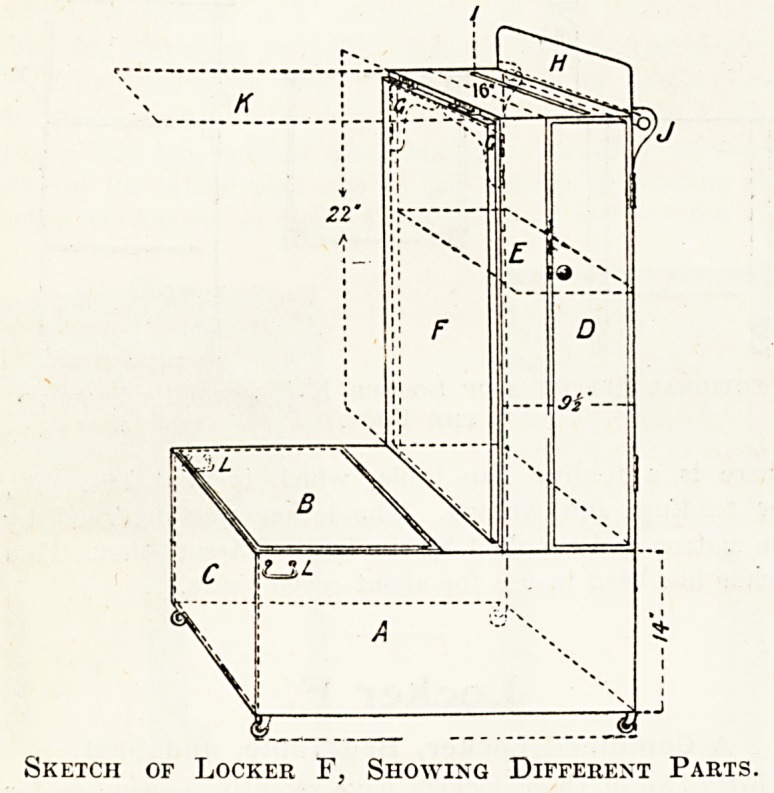


**Figure f12:**